# Effects of Nitrogen Application Strategies on Yield, Nitrogen Uptake and Leaching in Spring Maize Fields in Northwest China

**DOI:** 10.3390/plants14071067

**Published:** 2025-03-31

**Authors:** Ying Wang, Jingjing He, Zongyuan Gao, Ruliang Liu, Yu Hong, Fang Wang, Xinping Mao, Tianxiang Xu, Lina Zhou, Jun Yi

**Affiliations:** 1 Institute of Agricultural Resources and Environment, Ningxia Academy of Agro-Forestry Science, Yinchuan 750002, China; 2 School of Agriculture, Ningxia University, Yinchuan 750021, China; hejingjing116@163.com (J.H.);; 3 National Agricultural Environment Yinchuan Observation and Experiment Station, Ningxia Academy of Agro-Forestry Science, Yinchuan 750002, China; 4 Hubei Province Key Laboratory for Geographical Process Analysis and Simulation, Central China Normal University, Wuhan 430079, China

**Keywords:** spring maize, N leaching, inorganic N residue, N balance, N use efficiency

## Abstract

Nitrogen (N) is an essential nutrient for crop growth, as N fertilizer application regulates crop nitrogen uptake, affecting leaf photosynthetic rates, crop growth, and yield formation. However, both N deficiency and excess can reduce corn yields. Hence, optimizing the N fertilizer application strategy is crucial for crop production. In this study, a field plot trial with five N fertilization application strategies was conducted in the maize field from 2021 to 2022 in the Ningxia Yellow Irrigation District, Northwest China. These strategies contain zero N application rates (CK, 0 kg ha^−1^), the farmer practical N fertilizer application strategy (FP, 420 kg ha^−1^), the optimized N fertilizer application strategy (OPT, 360 kg ha^−1^), organic fertilizer and chemical fertilizer combination application (ON, 300 kg ha^−1^), and controlled-release N fertilizer and 33 urea application (CN, 270 kg ha^−1^). The maize yield and N balance under each treatment were investigated to propose the optimized N application strategy. The results showed that the CN treatment’s grain yield (15,672 kg ha^−1^) was the highest in both years, which was 109.97% and 8.92% higher than the CK and FP treatments, respectively. The apparent utilization rate and partial productivity of N fertilizer decreased with the increase in the N application rate. Also, the apparent utilization rate of N fertilizer in CN was 23.02%, 19.41%, and 13.02% higher than the FP, OPT, and ON, respectively. Applying controlled-release urea and organic fertilizers improved the physical and chemical properties of the soil, increased the organic matter content and soil fertility, and ultimately increased the spring maize yield. Meanwhile, the TN, NO_3_^−^-N, and NH_4_^+^-N concentrations in leaching water significantly correlated with the N application rate. With the extension of the maize growth period, the concentrations of TN, NO_3_^−^-N, and NH_4_^+^-N in leaching water gradually decreased. The N leaching amount in FP was the highest, while the CN was the lowest. The NO_3_^−^-N is the primary N leaching form, accounting for 46.78~54.68% of the TN leaching amount. Compared with the CN, the ON significantly increased the inorganic N content in the 0–40 cm soil layer, and it reduced the residual inorganic N content below 40 cm soil depths compared with FP and OPT treatments. Considering the relatively high spring maize yield and N utilization efficiency, as well as the relatively low N leaching amount and soil inorganic N residues, the ON and CN treatments with 270–300 kg ha^−1^ N application rate were the optimized N application strategies in the spring maize field in the study area.

## 1. Introduction

Maize (*Zea mays* L.) is an important food crop and feed source. It is also the world’s highest total-yielding food crop, with the global maize planted area reaching 197 million hectares (ha), second only to wheat [1]. Maize is currently the most widely cultivated and highest-yielding food crop in China, with the area planted with maize increasing from 15.20% of the total national crop area in 1995 to 25.68% in 2021, reaching 0.043 billion ha [2]. The Diversion of Yellow Irrigation District is one of the four major self-flowing ancient irrigation districts in China and a vital grain base in China [3]. In 2021, the maize planting area in Ningxia accounted for 31.24% of the total crop planting area in the region. Ningxia Yellow Diversion Irrigation Area is located inland in Northwest China. The Yellow River water diversion is mainly used for agriculture, and agricultural irrigation water accounts for more than 85% of the total water diversion. Due to the unique irrigation–-drainage system, the receding water returned to the Yellow River through drains and seepage is approximately 3 billion m^3^ annually. It was reported that the proportion of drainage diversion is approximately 40% of the irrigation amount, and the nutrients carried in the receding water pose a great risk to the water quality of the Yellow River upstream [4]. A study shows that the average input of pure nitrogen (N) in the process of maize cultivation in the Ningxia diversion area reached 408 kg ha^−1^ [5], significantly higher than the average amount of N applied in China (249 kg ha^−1^) [6], mainly due to the low nutrient content and sandy soil texture. The excessive N inputs and losses significantly impact the water quality, leading to the condition of the drainage ditch of the Ningxia diversion area mainly to the inferior class V [7]. It seriously affects the sustainable development of the regional agricultural economy and ecological environment. Consequently, the rational application of N fertilizer has become a key measure to balance economic and environmental benefits [8].

N is one of the most critical macronutrients required for plant growth. Adequate N fertilization is essential for efficient crop production and optimum yield [9]. Due to the high demand for agricultural products, the target yield will be set at the maximum yield level of N application. However, the over-application of N causes salinity stress that negatively affects metabolism, especially the N or carbon assimilation pathways, reflected by reduced growth indexes and yield [10]. Furthermore, the over-application of N increases the risk of N loss by runoff and leaching, which leads to deleterious effects on aquatic ecosystems through eutrophication [11]. The goal of sustainable N fertilizer management should be to improve the synchronization between crop N supply and crop N demand to limit N losses. The efficiency of N fertilizer use in current agricultural systems is closely linked to sustainable development, climate change, environmental quality, and food security issues. Crop growth is frequently exposed to various environmental stresses that result in considerable changes in metabolism, thus posing severe threats to yield and crop production. The results of previous studies have proved that traditional mineral N fertilizer application is not sufficient to meet the present environmental safety requirements, and the main techniques to improve the efficiency of N fertilizer use in maize are through optimization of N application, the application of controlled-release N fertilizers, and the formulation of organic fertilizers [12,13,14]. Among the different techniques, organic–inorganic blending synergistically increases growth at maximum grouting rate and maximum grouting rate by optimizing grouting characteristics, and organic N replaces 50% of inorganic N to maintain high maize yields while reducing NH_3_ and N_2_0 emissions and N loss [15,16].

As a vital component of chlorophyll, plant N levels are closely related to leaf senescence and photosynthesis [17]. During the reproductive growth stage, N availability prolongs the greenness of leaves, while N deficiency leads to premature leaf senescence [18]. Generally, the peak of N uptake in maize is at the spathe stage, and the application of adequate fertilizers at the spathe stage is essential for obtaining the highest efficiency of N uptake; however, the high height of maize plants at the spathe stage causes inconvenience in applying fertilizers and increases the demand for labor, which leads to the low efficiency of fertilizers at this stage in practice [19]. Controlled-release N fertilizer is a kind of fertilizer that uses a polymer coating to control the amount and release period of fertilizer nutrients, and by consistently matching the rate of N release with the pattern of crop N uptake, it can reduce the content of NO_3_^−^-N and NH_4_^+^-N in the deeper soils, and reduce the risk of nitrate N leaching to the deep soil layers [20]. Organic fertilizer is necessary to supplement the soil carbon source, maintain the soil organic carbon pool, and enhance soil water and fertilizer retention properties. Consequently, it has the advantages of increasing soil fertility, improving soil physicochemical properties, and causing less environmental pollution [21]. Single application of organic fertilizer is inefficient, promotes greenhouse gas emissions, and does not significantly increase yields [22]. Nitrate leaching in organic and inorganic integrated farming systems is significantly lower than in conventional farming systems, and increased soil organic carbon increases soil microorganisms, thereby converting nitrate, reducing soil nitrate pools, and nitrate leaching [23]. Long-term organic and inorganic inputs can significantly increase the content of soil organic carbon pools [24], increase dry matter and N accumulation in summer maize, improve seed yield and N use efficiency, reduce ammonia volatilization compared with urea alone, and reduce N_2_O emission compared with organic fertilizer alone [25,26]. Combined with the results of previous studies, we assumed that the physiological effects of different N application strategies on spring maize can be shown by yield. Although many studies evaluated the effects of the nitrogen fertilizer application strategy or fertilizer type on grain yield and nitrogen loss in the maize field, fewer were conducted in the Ningxia Yellow River irrigation area during the same period.

The objectives of this study were: 1, to compare the effects of different N application strategies on maize yield and N uptake and reveal its mechanism; 2, to quantify the N balance components under different N application strategies; 3, and to propose the optimal N application strategy in the study area.

## 2. Materials and Methods

### 2.1. Site Description

The experiment site was located at the field observation and experiment base of the Crops Research Institute of Ningxia Academy of Agriculture and Forestry, Wanghong Township, Yongning County. The area belongs to a typical continental arid climate, with multi-year average sunshine hours ranging from 2868 to 3060 h. The average temperature was 8.5~9.2 °C, and ≥10 °C cumulative temperature was 2900~3400 °C. The average annual precipitation was 180~200 mm, and the average annual water surface evaporation was 1600~2000 mm. The primary soil type in this area is anthropogenic-alluvial soil with coarse soil texture, characterized by a high sand content (>50%) and low clay content (<5%) [4].

The main soil formation processes in the irrigation and siltation soils of Ningxia Yellow River Irrigation Area are the soil maturation process caused by anthropogenic farming, the redox process caused by irrigation water and groundwater, and the surface physical matting process caused by sediment deposition of irrigation water, which is characterized by the soil formation environment of a high water table, a high salinity of the groundwater, and an arid climate with little rainfall and intense evaporation. The water table is only approximately 1 m in summer [27]. Soil bulk weight, porosity, and organic matter can well reflect the soil permeability and the environment for maize root growth; total salt affects maize growth and nitrogen fertilizer uptake; and total soil nitrogen and quick-acting nitrogen can well reflect the soil’s fundamental indexes about nitrogen as a way of determining the experimental scheme, which can contribute to the research findings (Table 1), and the analytical methods are referred to Soil Agro-chemical Analyses [28].

### 2.2. Experimental Design

Five fertilization application strategies were designed in the field plot trials, and each treatment was replicated thrice in a randomized block arrangement. The five treatments are zero N application rate (CK), the farmer practical N fertilizer application strategy (FP), the optimized N fertilizer application strategy (OPT), organic fertilizer and chemical fertilizer combination application (ON), and controlled-release N fertilizer and urea application (CN). FP treatment is the recommended N application rate of the local agricultural extension department, OPT treatment is the optimized recommended N application rate obtained from the previous research results of the research group, and ON and CN treatment are the optimized N application rates with a comprehensive consideration of N utilization and environmental effects of maize.

**Figure 1:**
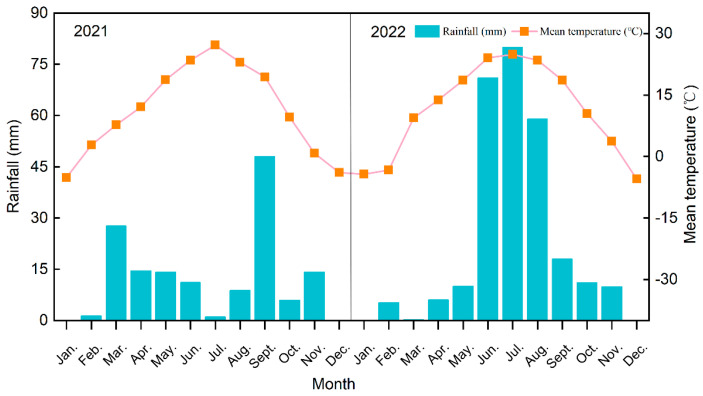
Monthly rainfall and temperature dynamics in the study area.

The field trial was carried out from April 2021 to October 2022. The plot area for each treatment was 50 m^2^ (10 m × 5 m), and the planting density was 71,500 ha^−1^. During the study period, 60% of N fertilizer was base fertilizer, and the remaining 40% was applied twice at the jointing and heading stages of maize. The top dressing was carried out on 18 June and 15 July in 2021, while it was carried out on 20 June and 20 July in 2022. Also, all phosphorus and potassium fertilizers are applied once as the base fertilizer. During the maize growth period, each plot was irrigated twice with the Yellow River water, and the irrigation amount was 150 mm each time. The irrigation was carried out the next day after each top dressing. The monthly rainfall and temperature dynamics are shown in Figure 1.

**Figure 2:**
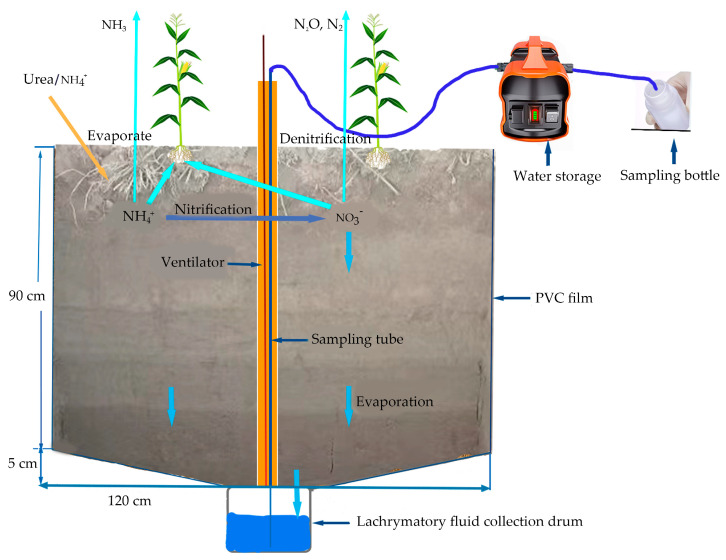
Loss-of-liquid collection device.

Figure 2 shows the field drench collection device. The soil needs to be excavated before the leaching barrel is buried. In order to ensure the reliability of the study results, the leaching solution was collected after the soil structure was stable in 2021. Collection of field eluent: an in situ collection method is adopted, an infiltration filter is built in each plot, and a water collecting bucket is installed. The infiltration filter is 120 cm long, 100 cm wide, and 90 cm deep. The excavated soil is stacked in various layers (i.e., 0–20, 20–40, 40–60, and 60~90 cm), and the trapezoid container is trimmed at the bottom to collect the leaching solution. Dig a small cylindrical section with a diameter of 45 cm and a depth of 35 cm at the center of the bottom, install a liquid collecting barrel with a volume of 50 L, cover the liquid collecting barrel with two layers of 0.25 mm nylon net, spread the washed gravel with a diameter of approximately 10 mm on the upper part of the nylon net with a thickness of 3 cm, and spread a layer of 0.25 mm nylon net on the upper part with a thickness of 2 cm. After the liquid collecting barrel is installed, the customized liquid collecting membrane is laid, so that the liquid collecting membrane forms a closed frame the same size as the pit and is backfilled in layers.

The maize variety of Xianyu 1225 was planted in this study. The N fertilizer was common urea with 46% N content, the phosphate fertilizer was superphosphate with 46% P_2_O_5_ content, the potassium fertilizer was potassium sulfate with 50% K_2_O content, and the controlled-release N fertilizer was resin-coated urea (produced by Anhui Maoshi New Fertilizer Co., Ltd., Hefei, China). The nutrient release period was 120 days when the cumulative release of N in water at 25 °C was more than 80%. Detailed fertilizer application information is shown in Table 2.

### 2.3. Sampling and Measurements

When maize is harvested, all ears are harvested in each plot, the number of ears is counted, the fresh weight of ears is weighed, and the average fresh weight of ears is calculated. According to the average fresh weight method, 20 ears are selected as samples and brought back to the laboratory for air drying and threshing to calculate maize grains’ seed yield and water content. Calculate the paid-in output with 14% water content. When maize was harvested, ten plants were randomly sampled from each plot and divided into straw and grain. After being immobilized at 105 °C for 20 min, they were dried at 70 °C to constant weight, which was used to determine the total N content in straw and grains. The total N content in various plant organs was determined by concentrated sulfuric acid digestion and the semi-micro Kjeldahl method [28].

Due to less precipitation and extensive evaporation in the experimental area, leachate in soil with a depth of 90 cm was only collected after each irrigation event. The water samples were collected three times a year after two topdressing irrigation and winter irrigation, and the eluent in the liquid barrel was collected within 5~7 days after each irrigation event. The volume of the eluent was measured, and the mixed water samples were taken to determine total N, ammonium N (NH_4_^+^-N), and nitrate N (NO_3_^−^-N) concentrations. The TN concentration was digested by alkaline potassium persulfate and determined by ultraviolet spectrophotometry. The concentrations of NO_3_^−^-N and NH_4_^+^-N were determined by TRAC CS-2000 continuous flow analytical (CFA).

The soil samples were sampled and analyzed from the irrigation and silting soil profile (0–100 cm). Soil bulk density and nutrients (total salt, organic matter, total N, NH_4_^+^-N, NO_3_^−^-N, alkali-hydrolyzable N) were determined. Soil samples of 0–20, 20–40, 40–60, 60–80, and 80–100 cm were collected after the annual spring maize harvest, and the contents of NH_4_^+^-N and NO_3_^−^-N in the soil were analyzed. Sampling 0–20 cm soil mixed samples, air dry them, and then pass them through a 20 mesh sieve to determine pH, organic matter, total N, alkali-hydrolyzable N, available phosphorus and potassium. A method for determining the contents of NO_3_^−^-N and NH_4_^+^-N comprises the following steps: weighing 10.00 g of fresh soil sample pass through a 2 mm sieve in a 100 mL plastic bottle, adding 50 mL of 1 mol L^−1^ KCl solution, shaking for 1 h, filtering, freezing the filtrate, and thawing before determination. Traccs-2000 continuous flow analytical (CFA) method was used for determination, soil pH was determined by potentiometry, organic matter was determined by potassium dichromate volumetric method-external heating method, total N was determined by Kjeldahl digestion method, soil alkali-hydrolyzable N was determined by alkaline diffusion method, available phosphorus was determined by Olsen method, and available potassium was determined by ammonium acetate leaching-flame spectrophotometry.

### 2.4. Calculations

Based on the above data, the apparent *N* use efficiency (NUE, %) and *N* partial productivity (NPP, kg kg^−1^) were calculated as follows:

(1) $$NUE(\%)=\frac{\left(U_{N}-U_{N0}\right)}{F_{N}}\times 100$$

where *U_N_* and *U_N_*_0_ are the total N uptake (kg ha^−1^) at the maturity stage with and without *N* fertilizer input, respectively, and *F_N_* is the amount of *N* fertilizer input.

(2) $$NPP=\frac{\left({GY}_{N\;}-{GY}_{N0}\right)}{F_{N}}$$

where *GY*_N0_ is the maize grain yield (kg ha^−1^) in CK treatments at the maturity period.

### 2.5. Statistical Analysis

The statistical software SPSS 22.0 was used for one-way analysis of variance (ANOVA) with the least significant difference (*LSD*) test at the 5% level.

## 3. Results

### 3.1. Effect of Optimized N Application on Maize Yield and N Absorption and Utilization of Spring Maize

**Figure 3:**
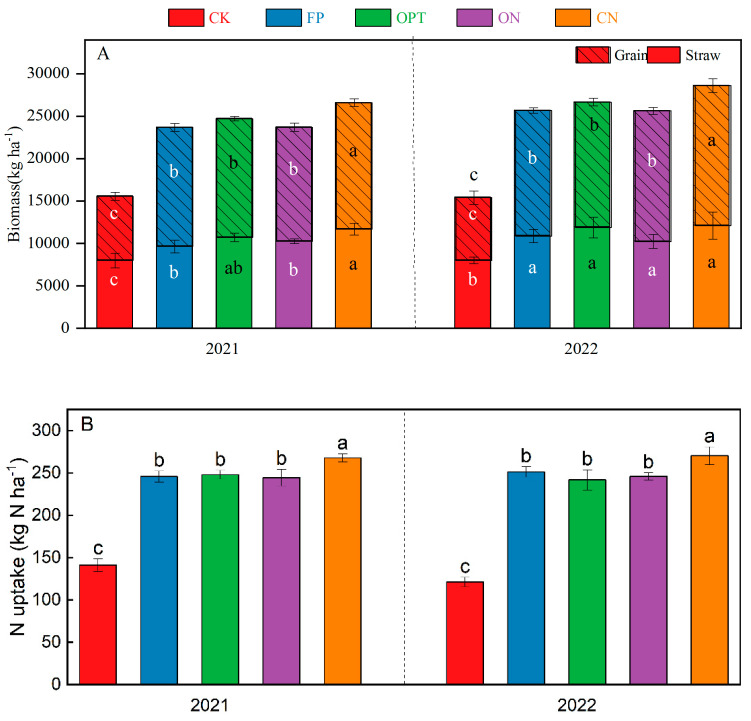
Above-ground biomass (**A**) and N uptake (**B**) of spring maize. Note: Additional lowercase letters represent significant differences (*p* < 0.05) in seed yield, straw biomass and N uptake under different N application strategies in the same period, and the error line represents the standard deviation.

Figure 3 shows that N application significantly increased the grain and straw yield. Among all the treatments, the grain yield of the CN was the highest in these two years, with an average grain yield of 15,672 kg·ha^−1^, which was 109.97% higher than the CK and 8.92% higher than the FP treatment. Due to the continuous non-application of N fertilizer, the grain yield of CK treatment in 2022 was lower than that in 2021, and the yield in other N treatments increased slightly. The average grain yield of FP, OPT and ON treatments was approximately 14,300 kg·ha^−1^, and there was no significant difference among these treatments (*p* < 0.05).

N application significantly increased the N accumulation above ground (Figure 3). The N absorption capacity of the CN treatment was the highest, with a two-year average of 269.07 kg·ha^−1^, which was 105.59% higher than the CK, and it increased by 9.90% and 8.30% compared with the OPT and FP, respectively. 

The NUE and NPP decreased with the increase in the N application rate (Table 3). The NUE and NPP of CN were significantly higher than those in other treatments (*p* < 0.05). Compared with FP, OPT and ON treatments, the NUE of CN treatment increased by 23.02%, 19.41% and 13.02%, respectively, and the NPP increased by 24.28%, 19.89% and 12.36%, respectively.

### 3.2. Effect of Optimized N Application on N Leaching Concentration and Amount

**Figure 4:**
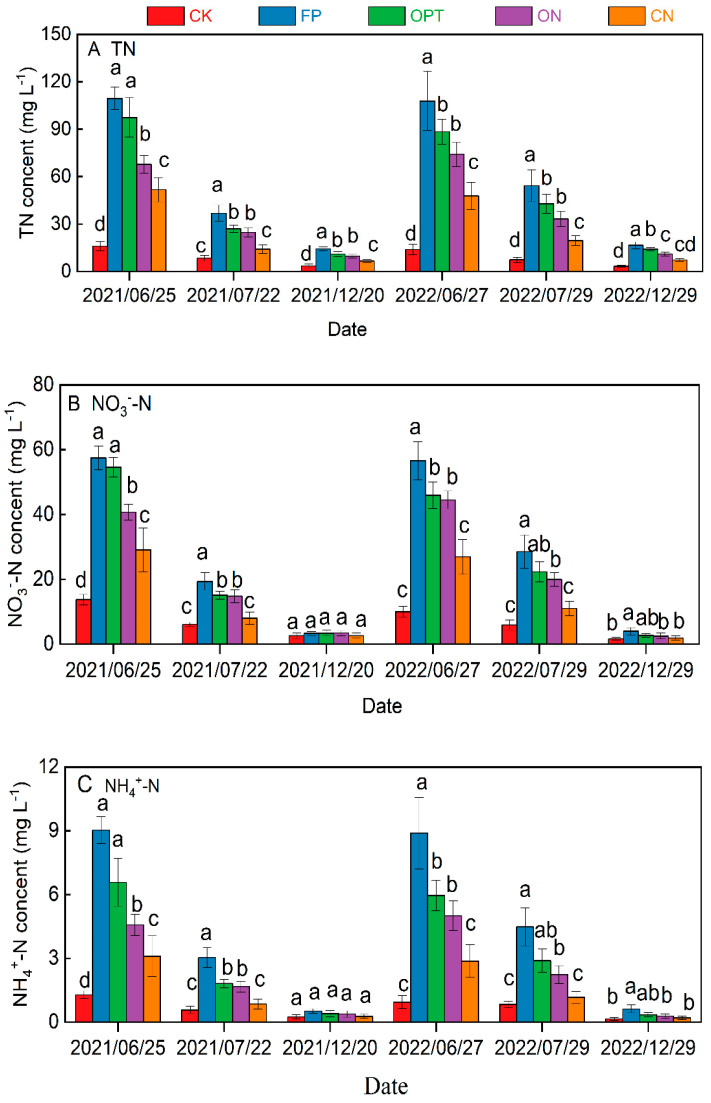
Concentration changes in TN (**A**), NO_3_^−^-N (**B**) and NH_4_^+^-N (**C**) in the leaching solution. Note: Additional lowercase letters represent significant differences (*p* < 0.05) in N leaching concentrations between different N application strategies during the same period, and the error line represents the standard deviation.

As shown in Figure 4, the TN, NO_3_^−^-N and NH_4_^+^-N concentrations in soil leachate significantly correlate with the N application level. With the extension of the maize growth period, the concentrations of TN, NO_3_^−^-N and NH_4_^+^-N in leachate gradually decreased. After the first topdressing of maize, the concentration of N in various forms in the leaching solution was the highest, and the concentration in leaching after winter irrigation was the lowest. The TN concentration of CK treatment was the lowest, ranging from 3.26 to 18.98 mg·L^−1^ in these two years. The highest TN concentration of FP treatment was 109.41 mg·L^−1^, with a range of 14.25~109.41 mg·L^−1^. Compared with FP treatment, the TN concentration of CN and ON treatment was significantly lower (*p* < 0.05). The two-year experiment showed that TN concentration in OPT, ON and CN treatments decreased by 14.50%, 34.67% and 54.15%, respectively, compared with FP treatment after the first topdressing. The TN concentration of the CN treatment was the lowest among all N application treatments, 29.83% lower than that of the ON treatment. After the second topdressing, the TN concentration of CK, FP, OPT, ON and CN treatments decreased by 47.01%, 58.05%, 62.42%, 59.22% and 66.19%, respectively. The N concentration in all optimized N application treatments was significantly lower than conventional treatment during the maize growth period, while there was no significant difference among N application treatments after winter irrigation. The changes in NO_3_^−^-N and NH_4_^+^-N concentrations in each treatment are similar to those in TN, and the NO_3_^−^-N concentration accounts for 52.50~79.25% of TN concentration. The two-year average NO_3_^−^-N concentration after the first topdressing in FP, OPT and ON treatments were 60.25, 48.81 and 38.13 mg·L^−1^, respectively. In contrast, the NH_4_^+^-N concentrations in all treatments were relatively low (<10 mg·L^−1^).

As seen from Table 4, the TN leaching amount increased with the increase in the N application rate. The average TN leaching loss in CK for two years was only 5.43 kg·ha^−1^, much lower than the TN N leaching amount in these application treatments (12.24~30.41 kg·ha^−1^). The leaching amount of TN in the FP treatment was the highest and much higher than in the N optimization treatment (*p* > 0.05). The TN leaching amounts of FP, OPT, ON and CN treatments were 30.41, 25.01, 15.24 and 12.25 kg·ha^−1^, respectively. Compared with FP treatment, the TN leaching amount of OPT, ON and CN treatments with optimized N application decreased by 17.76%, 49.88% and 59.94%, respectively. The leaching coefficients of FP, OPT, ON and CN treatments are 5.95%, 5.44%, 3.27% and 2.57%, respectively. NO_3_^−^-N is the main leaching form of N in each treatment, accounting for 48.07~56.05% of TN leaching amount, and NH_4_^+^-N leaching amount is less than 2.00 kg·ha^−1^ in each treatment, accounting for a small proportion of TN.

### 3.3. Effect of Optimized N Application on Inorganic N Content in 0–100 cm Soil Profile

**Figure 5:**
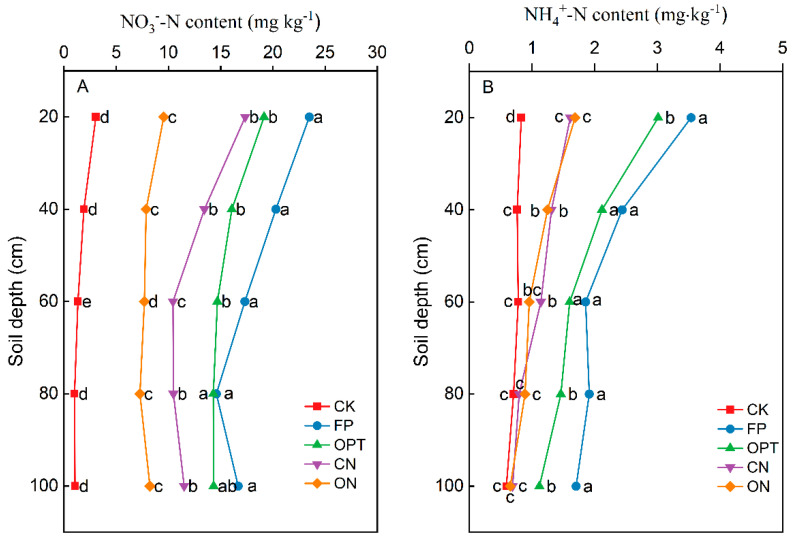
Distribution of inorganic N content in 0–100 cm soil profile after harvest. Note: Different lowercase letters after peer data indicate significant differences (*p* < 0.05).

Figure 5 showed that N fertilizer application significantly increased the residual inorganic N content (NO_3_^−^-N and NH_4_^+^-N) in 0–100 cm soil profile after spring maize harvest. The residual N contents increased with the increasing N application level, and the NO_3_^−^-N content in each treatment was significantly higher than that of NH_4_^+^-N. With the increase in soil depth, the overall change trend of soil NO_3_^−^-N decreased at 0–80 cm, slightly increased at 80–100 cm, and soil NH_4_^+^-N decreased at 0–100 cm. The NO_3_^−^-N content in all soil layers of CN was significantly lower than that of other treatments. The average NO_3_^−^-N content in 0–100 cm soil layers of CN was 47.92%, 38.79% and 23.93% lower than that of FP, OPT and ON treatments. Compared with CN treatment, the ON significantly increased the inorganic N content in 0–40 cm soil layers.

Meanwhile, ON significantly reduced residual inorganic N below 40 cm soil depth compared with FP and OPT treatments. In the 40–100 cm soil layer, the average NO_3_^−^-N content of ON treatment decreased by 32.24% and 25.14% compared with FP and OPT treatments. The NH_4_^+^-N contents in all treatments were low and decreased with the increase in soil depth. The NH_4_^+^-N contents in ON and CN treatments were approximately 50% lower than in FP and OPT treatments. Applying controlled-release N fertilizer and organic fertilizer significantly reduced the inorganic N percolation and reduced the residue of N fertilizer in the soil profile.

### 3.4. Effect of Optimized N Application on Soil Fertility in the Root Zone

**Figure 6:**
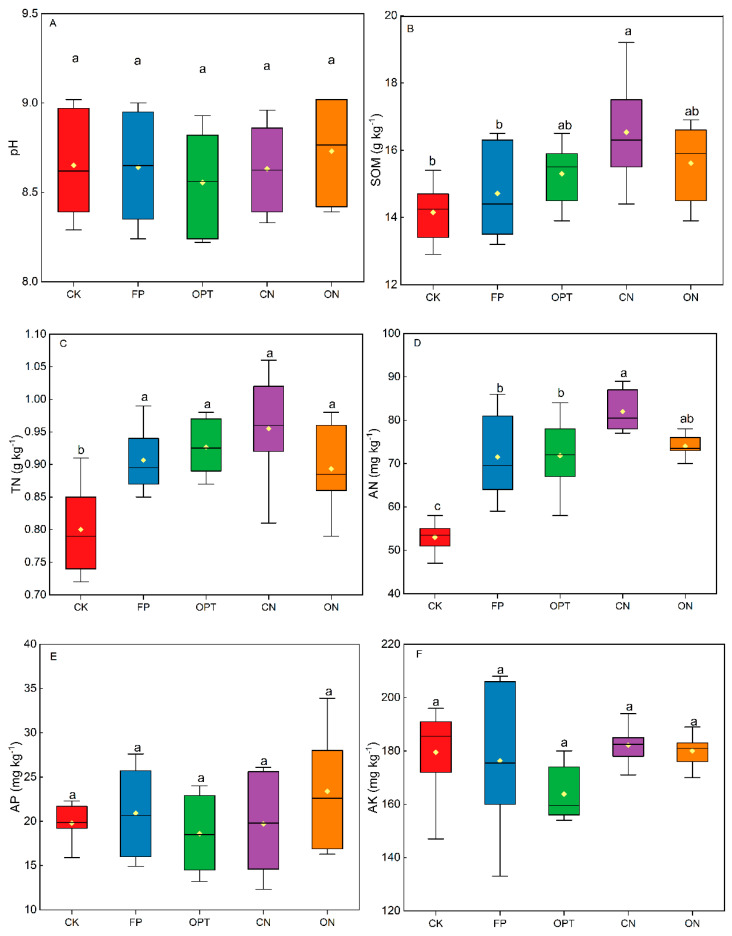
Differences in pH (**A**), organic matter content (**B**), total N content (**C**), alkali-hydrolyzable N content (**D**), available phosphorus content (**E**) and available potassium content (**F**) among different treatments. Note: Different lowercase letters after peer data indicate significant differences (*p* < 0.05).

There was no significant difference in soil pH among different fertilization treatments (Figure 6). The soil organic matter content in CN treatment was the highest (16.53 g kg^−1^), 16.82% and 12.34% higher than CK and FP treatments, respectively. There was no significant difference in soil organic matter content between OPT and ON. The total soil N content in all N application treatments was significantly higher than that in the CK, and the increased range of total N content in each fertilization treatment was between 11.67% and 16.84%, with the most extensive increment in CN treatment. N application significantly increased the alkali-hydrolyzable N content, which was in the order of CN > ON > OPT > FP > CK. There was no significant difference in available phosphorus and potassium contents among all treatments.

### 3.5. Effect of Optimized N Application on Apparent N Balance

The N balance in all treatments is shown in Table 5. During the maize growth period, irrigation and rainfall brought in 9.75 kg ha^−1^ N, and maize sowing brought in 0.49 kg ha^−1^ N. Table 5 shows that the apparent N loss increased with the increase in the N application rate, and the annual apparent N loss in all N application treatments was between 9.75 and 46.96 kg ha^−1^. The apparent N loss rate in FP, OPT, ON and CN treatments was 8.86%, 7.23%, 6.64% and 4.48%, respectively. The ON and CN treatments increased the crop N uptake, reduced the N leaching loss, improved the NUE and reduced the apparent N loss.

## 4. Discussion

### 4.1. Effects of the Optimizing N Application Strategy on Yield and N Fertilizer Utilization of Spring Maize

The application rate of N fertilizer has a significant effect on crop yield. The reasonable application of N fertilizer can improve the rate of grain filling and increase yield. Under excessive N application, the yield of maize is no longer significantly improved or even declined [29]. By applying N fertilizer, the chlorophyll content of leaves can be considerably increased, which is conducive to the utilization of light energy by leaves and, at the same time, improves the conversion of light energy to chemical energy [30]. Adequate N application can inhibit chlorophyll degradation, maintain cell viability, and prolong the greenness of plant leaves [31]. Insufficient or excessive N supply can lead to disorders in canopy structure, resulting in a decline in the crop population’s photosynthetic performance, further reducing yields [32].

At the same level of N fertilizer, N fertilizer setback can promote post-flowering dry matter accumulation and translocation to the kernel, increase the maximum growth rate of dry matter, and increase dry matter accumulation and kernel weight compared with conventional N application [33]. Nitrogen setbacks can delay the dry matter accumulation rate decrease in the late reproductive stage. The main reason is that nitrogen application can increase the content of superoxide dismutase and catalase in leaves [34]. Extending the duration of photosynthesis in leaves at the end of the reproductive stage increases the competition for organic matter in the grain “reservoir”, thus affecting the relationship between the “source” and the “reservoir”. The extension of the duration of leaf photosynthesis at the end of the reproductive period increases the competition for organic matter in the grain “pool”, thus affecting the coordination between the “source” and the “pool” [35]. The appropriate amount of nitrogen fertilizer can significantly increase the effective number of ears, the number of grains and the thousand-grain weight of winter wheat and spring maize [36,37,38], which is ultimately conducive to high yield. Controlled-release urea can continuously provide inorganic nitrogen to meet winter wheat’s mid- and late-growth needs, promote nitrogen uptake by plants, avoid the problem of labor for fertilizer follow-up, and significantly increase grain yield.

Organic fertilizer contains many beneficial bacteria and micronutrients required by crops, balances soil nutrients and releases elements in the fixed state of the soil [39,40]. Organic–inorganic fertilizer blending is vital to improve maize yield [41]. Blending organic fertilizer based on suitable nitrogen fertilizer transport can improve soil microbial and enzyme activity [42], improve soil physicochemical properties, and increase the content of organic matter and nutrients. It can enhance soil water and fertilizer retention in the surface layer. Organic and inorganic fertilizers can improve the water use efficiency of maize and nitrogen fertilizer utilization [43], which is particularly important for yield preservation in drought years. Some studies have concluded that controlled-release N fertilizers and ordinary urea have different yield effects on rice with different rates and application methods. For example, Fu et al. [43] pointed out that 50% controlled-release fertilizer and 50% ordinary nitrogen fertilizer applied at one time can better meet the nutrient demand of high-quality japonica rice at all stages of fertility and then obtain high yield.

The results of this study were similar to others [44,45], in which controlled-release N fertilizer mixed with conventional urea treatment with 270 kg ha^−1^ N had the highest yields, and compared with the traditional N application by the farmers (420 kg ha^−1^) N was reduced by 35.71% and maize kernel yield increased by 8.92%. The yield of conventional N application and organic–inorganic mixture treatments was maintained at 14,300 kg ha^−1^. As the N utilization efficiency decreased with the increase in N input, only approximately 30~50% of the applied N fertilizer was taken up by the crop, which was consistent with the results of the previous study [44]. It was found that the overuse of N fertilizer resulted in a significant decrease in soil C/N ratio, causing soil salinization, altering the composition, abundance, and function of soil microbial communities, and aggravating harmful plant-soil soil conditions [46]. Hence, controlling the amount of N fertilizer application is essential.

### 4.2. Effects of Optimized N Application Strategies on Inorganic N Leaching and Accumulation

N loss from farmland is a major driver of surface water pollution in China [47]. Excess N enters water bodies through surface runoff and subsurface leaching, exacerbating the risk of eutrophication. For every 10% increase in N application, the intensity of total N loss increases by approximately 2.82% [48], which brings severe problems of fertilizer waste and environmental pollution [49]. Precipitation and fertilizer application are the key drivers of N loss, especially the effect of heavy precipitation soon after fertilizer application [50]. Increased risk of nitrogen loss is also usually associated with asynchrony between nitrogen supply and crop demand, so improving the match between soil nitrogen supply and crop demand is an effective way to reduce nitrogen loss and efficiently use nitrogen fertilizer. Conventional treatment nitrogen pre-input proportion is too high. However, at this stage, due to the small individual plant, the nitrogen nutrient demand is relatively small, resulting in excessive soil inorganic nitrogen content in the pre-reproductive stage of rice, so that soil nitrogen seeps into the deeper layers of the soil under the action of gravitational water, which results in a large amount of nitrogen loss [51].

Compared with ordinary urea, controlled-release nitrogen fertilizer can significantly reduce the risk of soil nitrogen loss by extending the nitrogen supply time [52]. This study showed that during the spring maize cultivation period with limited rainfall, the flooding irrigation and N application were the main influences on N leaching loss. In contrast, the N leaching was very low in the field without N fertilizer application, and the leachate N amount increased with the increase in N application. The total N leaching from FP treatment was the highest (30.41 kg ha^−1^), and it was reduced by 15.18 and 18.17 kg ha^−1^ for ON and CN treatments, respectively. N leaching was dominated by NO_3_^−^-N, which accounted for 48.07~56.05% of TN, while the NH_4_^+^-N leaching amount was less than 2 kg ha^−1^ in all treatments. The highest NO_3_^−^-N leaching rate was 15.96 kg ha^−1^, observed in FP treatment, much higher than the NH_4_^+^-N leaching amount. These findings were consistent with the other studies [30], which showed that NO_3_^−^-N accounted for 50.6~82.4% of the total N in the leaching. The reason is that NH_4_^+^-N is readily adsorbed by soil colloids and fixed by a mineral lattice in soil. In contrast, the oxidation conditions of dry land soil are favorable for the formation and accumulation of NO_3_^−^-N, and it is easily soluble in water and hardly absorbed by colloid, which is the main form of N loss in dryland soil [30]. Due to the high N concentration in the leachate, reusing the leached N for irrigation in the groundwater and drainage ditch was beneficial for reducing nitrogen loss and water pollution.

Too much N accumulation in the soil profile will result in obvious N leaching in the following seasons [53]. Therefore, reducing N accumulation in the soil profile is the key to lowering N leaching in the future [54]. Previous studies have shown that effective N management, including fertilization techniques, appropriate amount and date of N application, can ensure cereal crops’ maximum yield and N efficiency [55,56]. The residual inorganic N in 0–100 cm soil profile increased with the N application level, and the NO_3_^−^-N content was significantly higher than the NH_4_^+^-N content in all treatments. Reducing the amount of N fertilizer application achieves the balance between crop demand and N supply [57], and reducing N fertilizer input is an effective measure to reduce N leaching [58]. When N fertilizer is supplied in excess, most of the N in soil and fertilizer is leached into the lower soil layer in the form of soluble NO_3_^−^-N and NH_4_^+^-N after rainfall and irrigation events, threatening groundwater safety [7]. The change tendency of soil inorganic N residue and apparent N loss is consistent, and both show a linear increase trend with the increase in the N application rate [59]. This study found that the NO_3_^−^-N content in all soil layers was significantly reduced when controlled-release N fertilizer was used, and the inorganic N content in soil was increased by 0–40 cm when organic and fertilizer was applied. The residual inorganic N below 40 cm was significantly reduced compared to farmers’ conventional N application treatment. The ON and CN treatments reduced the NO_3_^−^-N and NH_4_^+^-N contents in deep soil layers. This is mainly because organic fertilizer can improve soil structure, coordinate soil water, fertilizer, air and heat, and improve soil air permeability and its water and fertilizer conservation performance [14], while controlled-release fertilizer is consistent with the law of N absorption by crops, and the residue is reduced. It can also be seen that N applied to the soil will inevitably produce a certain amount of accumulation or loss. Therefore, in production practice, N application should try to control the excessive accumulation and loss of inorganic N to meet the demand of crops for N and promote the soil N supply capacity.

### 4.3. Effects of Optimizing N Application Strategies on Soil Fertility and N Apparent Balance

Nutrients in soil directly affect maize yield, as the organic carbon, N and phosphorus in soil are the key to determining crop yield [60]. Organic fertilizer increases soil organic matter’s supply and directly increases soil’s organic carbon content. Long-term combined application of organic and chemical fertilizers can increase soil N content, and the residual effect of organic fertilizer is continuously superimposed, which gives the soil a strong and lasting N supply capacity. This study showed that compared with conventional urea, long-term application of controlled-release urea and combined application of organic fertilizer significantly affected the improvement of soil organic matter, and the organic matter content increased by 6.12% and 12.34% for CN and ON, respectively. It was reported that organic carbon and TN in soil are highly compatible, and proper application of N fertilizer increased organic carbon content [61], thus promoting maize growth and soil nutrient balance [62]. The findings showed that the nutrient balance, soil nutrient content and crop fertilizer demand under various fertilizer treatments were vital in determining maize yield in a long-term field experiment. In addition, selecting the appropriate fertilizer type and applying the optimized amount was beneficial to achieve balanced fertilization in farmland [63]. This study showed that the lack of N fertilizer in CK treatment led to the lowest N content in the soil.

In contrast, farmers’ conventional N application rate leads to excess N, and the NUE decreases with the increase in the N application rate. The highest N uptake in CN treatment indicated that the soil’s N surplus can be indirectly reduced by matching the N absorption law of spring maize. The N leaching loss of spring maize and the excess N in the soil increased significantly due to farmers’ routine treatment, which quickly led to N loss by ammonia volatilization and N leaching, considerably affecting the agricultural ecological environment’s safety [3]. It was reported that the nitrogen fertilizer management pattern significantly affected ammonia volatilization losses, and a one-time basal application using a combination of regular and slow-release urea significantly reduced ammonia volatilization losses by 8.27% to 14.22% [64]. Another study found that 20% N reduction followed by 20% N replacement with organic fertilizer did not result in significant yield differences compared to the customary N application, but reduced N_2_O emissions by 31.72% [65]. These results indicated that the combined application of organic and inorganic fertilizers and mixed application controlled-release and conventional urea were suitable fertilization methods in this study area, which enhanced maize yield and reduced N loss simultaneously of spring maize fields in the irrigation area of Yellow River Diversion.

## 5. Conclusions

This experiment investigated the yield and N leaching loss characteristics of spring maize in the diversion irrigation area based on a large field experiment. A general method of organic–inorganic matching is proposed to determine the N fertilizer application strategy for high crop yield and low environmental pollution. We optimized the N application technology model for environmentally friendly maize. The results of this paper are as follows:

(1) The conventional N application 420 kg ha^−1^ strategy has additional risks, including the potential loss of yield, equivalent total applied N but lower yield, nitrate N and ammonium N residues in the soil significantly higher with the increase in N application, and the tendency to continue to migrate to the deeper soil layers, increasing the leaching loss.

(2) Optimization of N application treatments in the use of controlled-release N fertilizers and with the application of organic fertilizers after the average yield of maize no longer increased with the increase in the amount of N fertilizers, indicating that the fertilizer combination played the agricultural optimal N application strategy, N fertilizers 270–300 kg ha^−1^ dosage of N fertilizers to provide sufficient N for optimal maize yields, in the absence of a reduction in the yield of spring maize conditions, and significantly reduced the loss of mineral N in the maize season to the deeper layers of the soil body of the leaching.

(3) Application of controlled-release urea and organic fertilizers with a tendency to continue to migrate to the deep soil body. Application of controlled-release urea and dosed organic fertilizers improved soil physical and chemical properties, increased soil fertility, and promoted soil nutrient balance

This study has some limitations that should be improved in future studies. First, this study only identified recommended N application strategies, and further research should measure the effects of N application on greenhouse gas emissions such as N_2_O as well as the direct effects of climate change on yields and water quality and their linkage effects on key N application strategies deserves to be fully assessed.

## Figures and Tables

**Table 1:** The basic soil properties in the study area.

Soil Depth (cm)	Bulk Density (g cm^−3^)	Total Porosity (%)	Total Salt (g kg^−1^)	Organic Matter (g kg^−1^)	Total N (g kg^−1^)	Available N (mg kg^−1^)
0–20	1.36 ± 0.11 a	48.7 ± 3.90 a	0.49 ± 0.04 a	13.74 ± 1.1 a	1.01 ± 0.08 a	38.66 ± 3.09 a
20–40	1.36 ± 0.14 a	48.8 ± 4.88 a	0.40 ± 0.04 b	8.71 ± 0.87 b	0.85 ± 0.09 b	26.98 ± 2.70 b
40–60	1.53 ± 0.23 a	42.3 ± 3.35 a	0.39 ± 0.06 bc	5.26 ± 0.79 c	0.40 ± 0.06 c	25.12 ± 3.70 b
60–80	1.64 ± 0.11 a	39.0 ± 2.73 a	0.35 ± 0.02 bc	4.41 ± 0.31 cd	0.31 ± 0.05 c	24.31 ± 3.70 b
80–100	1.44 ± 0.26 a	45.4 ± 4.17 a	0.31 ± 0.06 c	3.15 ± 0.57 d	0.29 ± 0.05 c	23.58 ± 4.27 b

Note: Different lowercase letters in the same column indicate significant differences (*p* < 0.05).

**Table 2:** Fertilization application amount in different treatments (kg ha^−1^).

Treatment	Organic Fertilizer	Urea N	Control-Released Uera N	P_2_O_5_	K_2_O
CK	0	0	0	105	45
FP	0	420	0	105	45
OPT	0	360	0	105	45
ON	4500	300	0	105	45
CN	0	135	135	105	45

**Table 3:** N use efficiency (NUE) and N partial productivity (NPP) under different treatments.

Treatment	NUE/%			NPP/kg·kg^−1^		
	2021	2022	Average	2021	2022	Average
FP	24.89 ± 1.58 d	30.46 ± 1.46 c	27.68 ± 1.27 d	33.34 ± 1.07 d	35.18 ± 0.73 d	34.42 ± 1.17 d
OPT	29.65 ± 1.44 c	32.91 ± 33.1 c	31.28 ± 1.88 c	38.84 ± 0.69 c	40.95 ± 1.27 c	38.81 ± 1.52 c
ON	34.45 ± 3.27 b	40.90 ± 1.40 b	37.67 ± 0.98 b	44.73 ± 1.66 b	51.19 ± 1.36 b	46.34 ± 0.71 b
CN	46.92 ± 1.79 a	54.46 ± 3.89 a	50.69 ± 2.16 a	55.09 ± 1.67 a	61.01 ± 3.13 a	58.70 ± 1.92 a

Note: Different lowercase letters after each year’s data in the same column indicate significant differences (*p* < 0.05).

**Table 4:** Leaching amount of TN, NO_3_^−^-N and NH_4_^+^-N (kg·ha^−1^).

Treatment	TN		NO_3_^−^-N		NH_4_^+^-N	
	2021	2022	2021	2022	2021	2022
CK	5.96 ± 0.49 e	4.96 ± 0.84 d	3.06 ± 0.68 d	2.75 ± 0.45 e	0.45 ± 0.04 c	0.37 ± 0.07 c
FP	30.04 ± 2.56 a	30.77 ± 1.20 a	15.77 ± 1.77 a	16.15 ± 1.25 a	1.82 ± 0.55 a	1.76 ± 0.34 a
OPT	25.79 ± 3.23 b	24.23 ± 2.62 b	14.75 ± 1.51 a	13.29 ± 1.21 b	1.71 ± 0.31 a	1.42 ± 0.33 ab
ON	16.48 ± 1.23 c	13.99 ± 01 c	9.10 ± 0.84 b	7.75 ± 0.78 c	1.38 ± 0.18 ab	1.08 ± 0.34 b
CN	12.22 ± 0.65 d	12.27 ± 1.15 c	6.65 ± 1.06 c	5.12 ± 0.83 d	1.09 ± 0.13 b	0.92 ± 0.18 b

Note: Different lowercase letters after each year’s data in the same column indicate significant differences (*p* < 0.05).

**Table 5:** N balance in 0–100 cm depth soil profile under different treatments (kg ha^−1^).

Items	CK	FP	OPT	ON	CN
Total N input	175.54	595.54	535.54	475.54	445.54
Fertilizer	0.00	420.00	360.00	300.00	270.00
Irrigation and precipitation	9.75	9.75	9.75	9.75	9.75
Seed	0.49	0.49	0.49	0.49	0.49
Soil mineral N before transplant	122.98	122.98	122.98	122.98	122.98
Apparent N mineralization	42.81	42.81	42.81	42.81	42.81
Total N input	175.54	595.54	535.54	475.54	445.54
Total N output	175.54	595.54	535.54	475.54	445.54
Maize plant N uptake	131.2	248.45	244.83	245.21	269.07
Soil mineral N after harvest	34.59	300.13	254.93	200.67	154.62
Apparent N loss	9.75	46.96	35.78	29.66	21.85

## Data Availability

The original contributions presented in this study are included in the article. Further inquiries can be directed to the corresponding author.
